# Proteasome Augmentation Mitigates Age‐Related Cognitive Decline in Mice

**DOI:** 10.1111/acel.14492

**Published:** 2025-02-13

**Authors:** Danitra Parker, Kanisa Davidson, Pawel A. Osmulski, Maria Gaczynska, Andrew M. Pickering

**Affiliations:** ^1^ Department of Integrative Biology and Pharmacology The University of Texas Health Science Center at Houston Houston Texas USA; ^2^ Department of Molecular Medicine UTHealth San Antonio San Antonio Texas USA; ^3^ Institute on Aging The University of Texas Health Science Center at Houston Houston Texas USA

**Keywords:** aging brain, mice, proteasome, proteostasis

## Abstract

The aging brain experiences a significant decline in proteasome function. The proteasome is critical for many key neuronal functions including neuronal plasticity, and memory formation/retention. Treatment with proteasome inhibitors impairs these processes. Our study reveals a marked reduction in 20S and 26S proteasome activities in aged mice brains, including in the hippocampus, this is driven by reduced functionality of aged proteasome. The decline in proteasome activity is matched by a decline in 20S proteasome assembly. In contrast, 26S proteasome assembly was found to increase with age, though 26S proteasome activity was still found to decline. Our data suggests that age‐related declines in proteasome activity is driven predominantly by reduced functionality of proteasome rather than altered composition. By overexpressing the proteasome subunit PSMB5 in the neurons of mice to increase the proteasome content and thus enhance its functionality, we slowed age‐related declines in spatial learning and memory. We then showed acute treatment with a proteasome activator to rescue spatial learning and memory deficits in aged mice. These findings highlight the potential of proteasome augmentation as a therapeutic strategy to mitigate age‐related cognitive declines.

## Graphical abstract

Proteasome function declines in the brain with age. Genetic tools to increase proteasome levels reduce age‐related spatial memory deficits. Acute treatment with proteasome activators rescue age‐related spatial memory deficits.

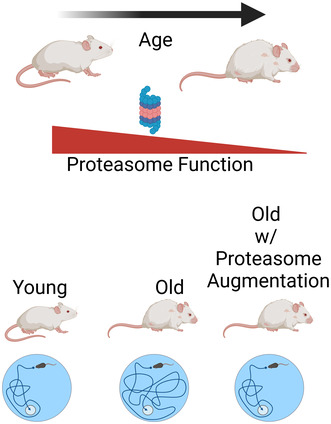

## 1 Introduction

Declines in proteasome function are a robust feature of aging reported in a wide range of tissues in mice (Bardag‐Gorce et al. 1999; Keller, Hanni, and Markesbery 2000; Rodriguez, Gaczynska, and Osmulski 2010), including the nervous system (Keller, Hanni, and Markesbery 2000). Similar declines are observed in the heads of fruit flies (Munkacsy et al. 2019) and the brains of killifish (Kelmer Sacramento et al. 2020). This decline correlates with increased levels of oxidized and polyubiquitinated proteins (Hamazaki and Murata 2024; Petropoulos et al. 2000; Rai et al. 2021).

## 2 Results

**FIGURE 1:**
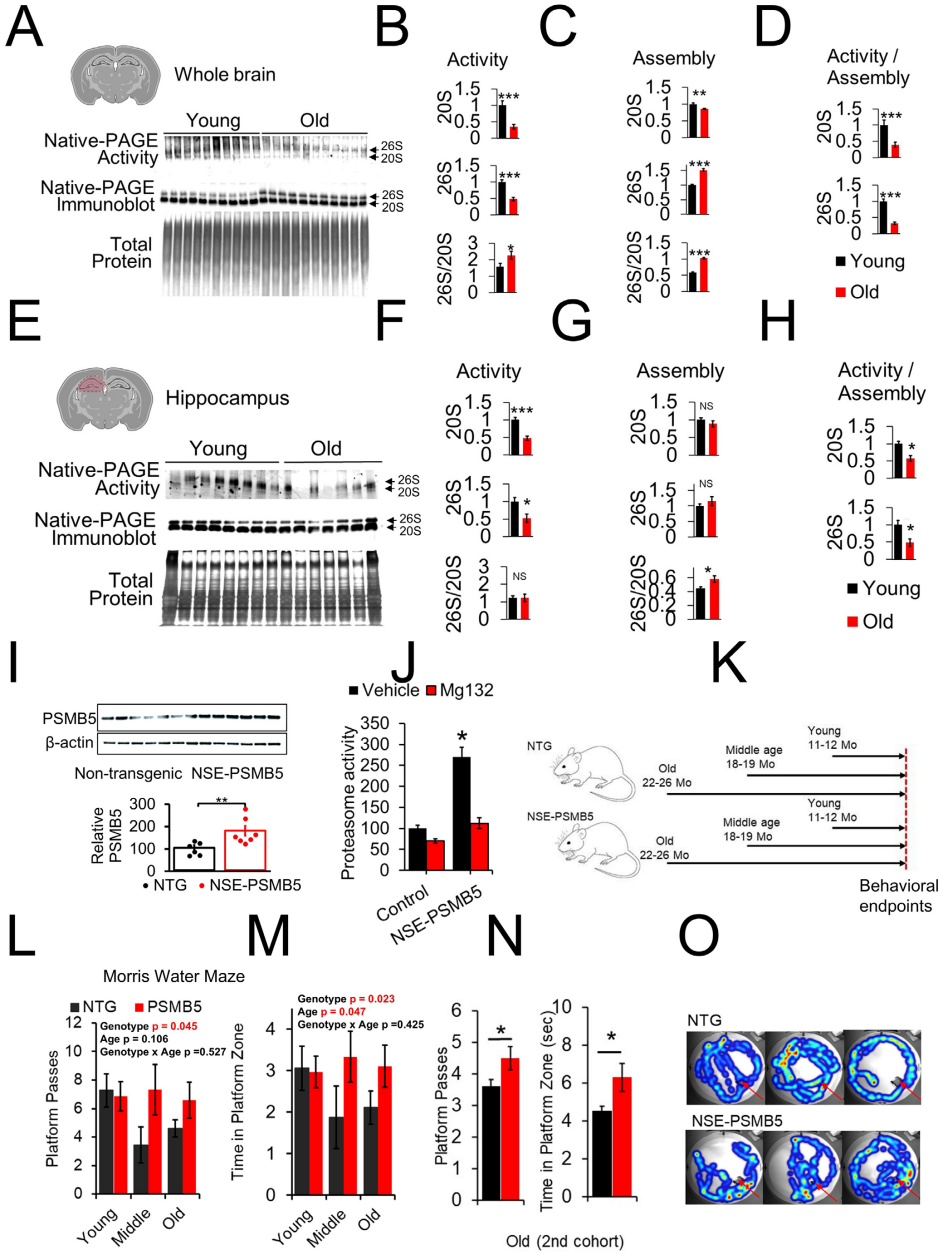
Neuronal PSMB5 overexpression mitigates age‐related cognitive decline in mice. (A) Native‐PAGE blot of whole‐brain samples from young (12 ± 1 Mo, *N* = 10) and old (24 ± 2 Mo, *N* = 11) mice showing active proteasome cores (top), proteasome assembly via anti‐PSMB5 native‐PAGE blot (middle), and total protein by silverstain (bottom). (B–D) Quantification of proteasome activity and assembly. (E–H) Native‐PAGE blot and quantification of hippocampus isolates from young (*N* = 8) and old (*N* = 7) mice. (I–J) PSMB5 immunoblot normalized to β‐actin and proteasome activity in 3‐MO mice (*N* = 5–7), including MG132 treatment. (K) Experimental design with cohorts for young, middle‐aged, and old mice. (L–M) Morris water maze results for NSE‐PSMB5 mice compared to controls *N* = 11–15 young, 3–7 Middle age, and 9–10 old. (N) Morris water maze results in second cohort *N* = 11–12 old. (O) Representative heatmaps from probe trials. Significance: ***p < 0.001, **p < 0.01, *p < 0.05, NS = not significant.

We examined proteasome activity and assembly in whole brains from young (12‐MO) mice, compared to old (22‐26‐MO) mice (Figure 1A). Activity was measured using a Native‐PAGE activity assay with a fluorescent proteasome activity probe MV151 as a measure of free active proteasome centers (Verdoes et al. 2006). We observed a significant decline in both 20S and 26S proteasome active centers with age (70% and 50% decline respectively) (Figure 1A,B).

We assessed proteasome assembly using Native‐PAGE immunoblotting and observed a modest decline in 20S proteasome assembly with age. Strikingly, we noted a pronounced 50% increase in 26S proteasome assembly with age, resulting in a higher 26S‐to‐20S assembly ratio (Figure 1C). This contrasts with previous reports of reduced 26S assembly in aging (Dasuri et al. 2009; Vernace et al. 2007). The discrepancy may arise from differences in the age of the “young” animals used. Earlier studies used 3‐MO mice (Dasuri et al. 2009), whereas we used 12‐MO. Given the elevated protein translation rates in the first 3–6 months of life (Kim et al. 2023; Ward and Richardson 1991), this early increase in 26S proteasome levels may account for the variation. Despite increased 26S assembly, we observed a 70% decline in the functionality of both 20S and 26S proteasomes, suggesting age‐related dysfunction arises from impaired proteasome activity, not reduced levels/assembly (Figure 1D).

We examined age‐related changes in proteasome function in the hippocampus, a region critical for spatial learning and memory. Similar to whole‐brain findings, both 20S and 26S proteasome activities were reduced with age (Figure 1E,F). While trends for decreased 20S assembly and increased 26S assembly were observed, these were not statistically significant. However, the 26S‐to‐20S assembly ratio was significantly elevated, though less pronounced than in the whole brain (Figure 1G). Notably, the ratio of proteasome activity to assembly for both forms declined with age (Figure 1H), indicating that proteasome functionality diminishes in the hippocampus with aging.

The proteasome is essential for neuronal processes like synaptic plasticity, dendritic spine growth, and memory formation. Proteasome inhibitors disrupt these functions (Davidson and Pickering 2023; Hamilton et al. 2012; Lopez‐Salon et al. 2001). Thus, we hypothesize that age‐related proteasome dysfunction may contribute to cognitive decline in the aging brain.

To test if preserving proteasome function alleviates age‐related cognitive deficits, we used a genetic approach to enhance proteasome assembly. Overexpression of the rate‐limiting proteasome subunit PSMB5 boosts proteasome activity (through increased number of proteasomes) in cell culture and invertebrates, and extends lifespan in worms and flies (Chondrogianni et al. 2015, 2005; Munkacsy et al. 2019; Nguyen et al. 2019). In flies, neuronal PSMB5 overexpression delays age‐related learning and memory decline (Munkacsy et al. 2019). We developed a transgenic NSE‐PSMB5 mouse model with neuronal‐specific PSMB5 overexpression (Chocron et al. 2022), which enhances brain proteasome activity and assembly (Figure 1I,J). Our prior study demonstrated its protective capacity against Alzheimer's pathology (Chocron et al. 2022).

To assess proteasome augmentation's effect on brain aging, we studied NSE‐PSMB5 mice and controls across three age groups: young (12 ± 1 months), middle‐aged (18 ± 1 months), and old (24 ± 2 months) (Figure 1K; Chocron et al. 2022).

**FIGURE 2:**
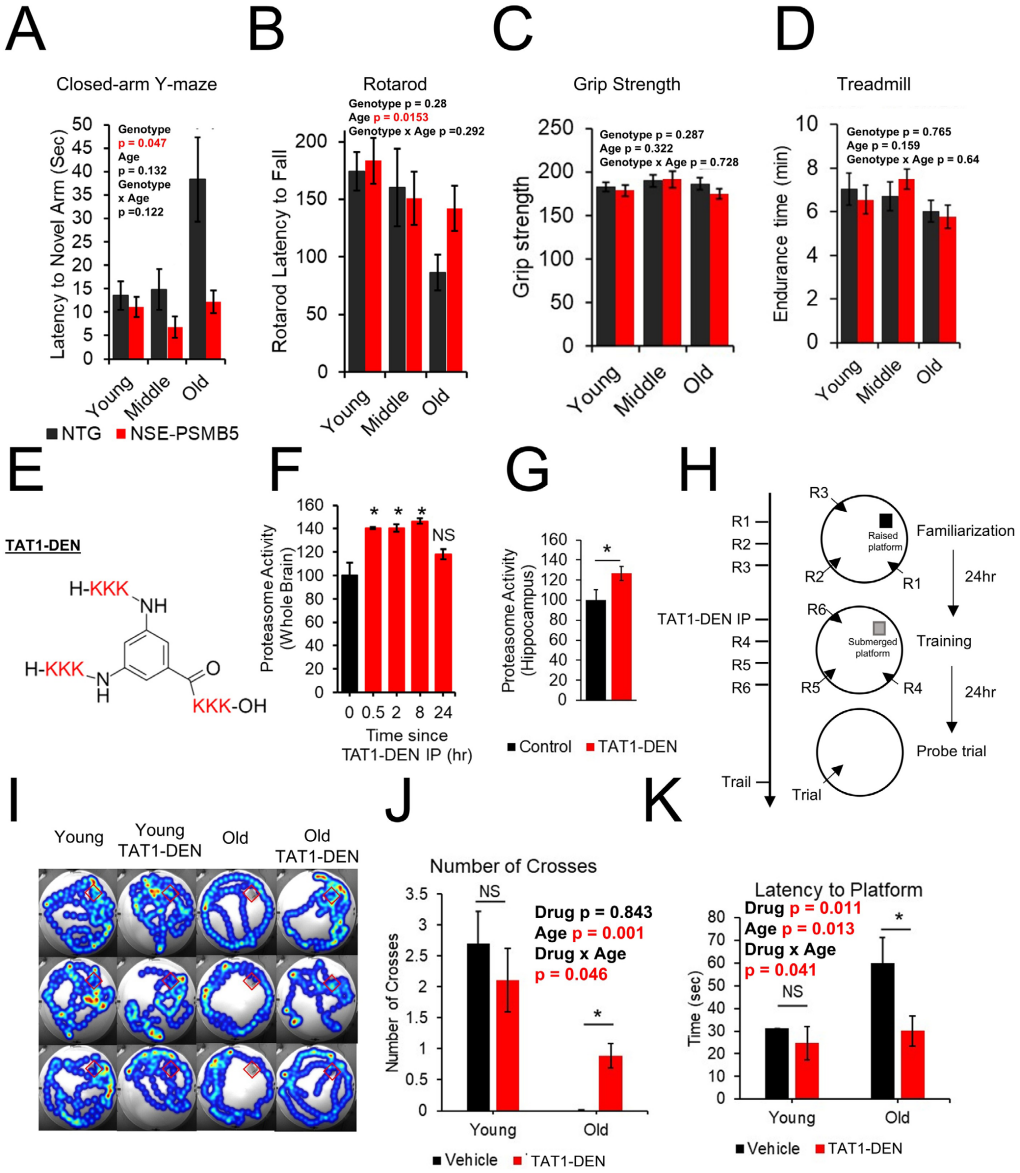
Acute treatment with proteasome agonist TAT1‐DEN rescues age‐related cognitive deficits. (A) Closed‐arm Y‐maze: Latency to enter the novel arm, comparing NSE‐PSMB5 and controls. (B) Rotarod: Latency to fall on day 2 of training. (C) Grip strength. (D) Treadmill maximum speed and endurance time. Animal numbers for A–D *N* = 11–15 young, 3–7 Middle age, and 9–10 old. (E) Structural schematic of TAT1‐DEN. (F–G) Proteasome activity in brain and hippocampal lysates after TAT1‐DEN injection *N* = 2–4. (H) Water maze design modified from (Artinian et al. 2008). (I) Representative heatmaps of probe trial (J–K) Probe trial results: Platform zone crosses and latency to reach zone., *N* = 18–19 young, 13–14 old. *p < 0.05, NS = not significant.

Using the Morris water maze, we observed improved spatial learning and memory in NSE‐PSMB5 overexpressing mice, with increased platform passes and time in the platform zone. These improvements were evident in middle‐aged and old cohorts, but not in young animals (Figure 1L,M). To confirm these findings, we repeated the assay in a larger cohort of old mice, which reproduced the improvements (Figure 1N,O). Similarly, in a closed‐arm Y‐maze assay, NSE‐PSMB5 mice showed enhanced spatial memory, particularly in the old‐age group (Figure 2A). These results suggest that PSMB5 overexpression mitigates age‐related cognitive decline in spatial learning and memory.

To assess whether cognitive improvements were linked to physical function, we tested rotarod, grip strength, and treadmill performance. Rotarod performance declined with age, with a non‐significant trend for improvement in older PSMB5‐overexpressing mice (Figure 2B). Grip strength showed no changes with age or transgene (Figure 2C). Treadmill performance showed a non‐significant age‐related decline and no significant transgene effect (Figure 2D).

We next investigated if transient augmentation of proteasome function via treatment with a proteasome activating compound could rescue age‐related cognitive deficits. We developed a set of proteasome activating peptidomimetics which show a robust ability to enhance both 20S and 26S proteasome function in vivo and in vitro, a detailed characterization is reported in our prior publications (Chocron et al. 2022; Osmulski et al. 2020). We recapitulated our prior finding that our lead TAT1‐DEN can enhance 20S proteasome function in the brains of mice under IP injection (Figure 2E,F). We further demonstrated capacity of our lead to enhance proteasome function in the hippocampus (Figure 2G). Previous studies have shown acute treatment with proteasome inhibitors to produce deficits in long term memory formation and retrieval (Artinian et al. 2008; Lee et al. 2008; Lopez‐Salon et al. 2001). Our running hypothesis is that age‐related declines in proteasome function may produce similar deficits. We thus hypothesized that acute treatment with a proteasome activator might rescue age‐related deficits in memory formation. To test this hypothesis, we employed ‘young’ (12‐MO) mice alongside ‘old’ (24‐month‐old) mice each IP injected with our proteasome agonist.

Animals were tested for spatial learning and memory using a modified Morris water maze. Our design, based on a modified design by (Artinian et al. 2008), included maze familiarization (day 1), proteasome activator (TAT1‐DEN) injection and training with a submerged platform (day 2), and a probe trial (day 3), (Figure 2H). Aged mice showed memory deficits, with fewer platform zone crosses and increased latency. While TAT1‐DEN had no effect on young mice, it significantly improved performance in aged mice (Figure 2J,K).

## 3 Discussions

In conclusion, we demonstrate that aging is linked to significant declines in proteasome functionality, driven by reduced proteasome activity rather than changes in expression or assembly. Enhancing proteasome levels and assembly in the nervous system via overexpression of a rate‐limiting proteasome subunit mitigates age‐related declines in spatial learning, memory, and neuro‐muscular function, with no impact on physical function. Additionally, acute treatment with a proteasome activator rescues cognitive deficits, highlighting the potential of both acute and prolonged proteasome modulation in reversing age‐related cognitive impairments.

This finding progresses on prior work where acute treatment of proteasome inhibitors have been shown to produce deficits in contextual and spatial learning and memory (Artinian et al. 2008; Lopez‐Salon et al. 2001; Rodriguez‐Ortiz et al. 2011). This is thought to be driven by engagement of the proteasome system in a range of processes critical to neuronal function including in long‐term potentiation, synaptic plasticity, as well as dendritic spine growth and stability (Davidson and Pickering 2023; Hegde 2010). Our findings also align with prior work implicating proteasome dysfunction in Alzheimer’s disease and showing that proteasome augmentation can alleviate its pathology (Chocron et al. 2022; Mladenovic Djordjevic et al. 2021). Together, our work underscores the central role of proteasome dysfunction in driving cognitive decline with aging and offers promising strategies for therapeutic intervention through proteasome enhancement.

## Author Contributions

Experiments: A.M.P., K.D., D.P. Compound development: P.A.O., M.G. Analysis and Writing: A.M.P., M.G., P.A.O.

## Conflicts of Interest

A.M.P., M.G., P.A.O. are inventors on a patent application related to this work filed by The University of Texas Health Science Center at San Antonio (HSC1567, filed 13 September 2019). The authors declare no other conflicts of interest.

## Supporting information


Appendix S1.

## Data Availability

The data that support the findings of this study are available from the corresponding author upon reasonable request.
