# Supplementary material for: Proteasome Augmentation Mitigates Age‐Related Cognitive Decline in Mice
Source: Aging Cell. 2025 Feb 13;24(3):e14492. doi: 10.1111/acel.14492 (PMC11896255; doi:10.1111/acel.14492)
Supplement: Supplementary file 1 — Appendix S1. [file ACEL-24-e14492-s001.pdf]

SUPPLEMENTAL EXPERIMENTAL PROCEDURES

NSE-PSMB5

A full-length mouse PSMB5 plasmid was used (MR203485, Origene). PSMB5 was excised to remove the Myc-DDK tag then cloned into the ShuttleNSE empty vector (50958, Addgene) adjacent to the NSE promoter. The NSE-PSMB5 region was excised and microinjected into (C57BL/6 X SJL) F2 mouse eggs at the University of Michigan Transgenic Animal Model Core. Mice were then bred into a C57BL/6J background for three generations. To control for the mixed backgrounds of mice, all experimental comparisons were made between littermates.

Sample lysis

Tissue flash frozen with liquid nitrogen and was powdered via pestle and mortar. Powder was weighed and proteasome activity buffer added proportionate to tissue weight. The reaction buffer consisted of 50 mM tris-HCl (pH 7.8) supplemented with 1 mM MgCl2, 0.5 mM ATP, and 1 mM DTT. Mechanical lysis was performed on ice via an electronic pestle. Samples were centrifuged and supernatant transferred to new tubes

Plate Based proteasome activity assays

Assays were initiated by the addition of 50 µM Suc-LLVY-AMC. For some assays, 1 µM proteasome inhibitor, MG132, was added 5 min before incubation with Suc-LLVY-AMC. The reaction was carried out with readings at 5-min intervals for a period of 4 hours at 37°C. Kinetic changes in fluorescence were monitored through recordings every 5 minutes for a 2hr period at 37ºC in a spectrophotometer at 380 nm/460 nm

Native PAGE MV151 assay

Supernatant was incubated with 1 µM proteasome probe MV151 for 1 hour at 4°C and then run on a 10% tris-glycine nondenaturing polyacrylamide gels (Bio-Rad) in Native Gel Buffer (diluted from 20×; Life Technologies) supplemented with 5 mM MgCl2, 1 mM DTT, and 0.5 mM ATP to maintain proteasome assembly. Gels boxes were surrounded by ice and run in a 4°C refrigerator at 100 V for 1 hour, followed by an additional 3 hours at 250 V. MV151 labelling was then measured by fluorescence under UV and ImageQuant 4000 (GE Healthcare), using 312-nm excitation and measuring emissions between 585 and 625 nm.

Native PAGE Immunoblot

Supernatant was run on a 10% tris-glycine nondenaturing polyacrylamide gels (Bio-Rad) in Native Gel Buffer (diluted from 20×; Life Technologies) supplemented with 5 mM MgCl2, 1 mM DTT, and 0.5 mM ATP to maintain proteasome assembly. Gels boxes were surrounded by ice and run in a 4°C refrigerator at 100 V for 1 hour, followed by an additional 3 hours at 250 V. The gel was incubated in running buffer containing 5% SDS, after which transfer and detection were performed following standard procedures.

Study approval

All mouse studies performed were approved by the Institutional Animal Care and Use Committee at the University of Alabama at Birmingham (protocol 22179; Pickering, PI).

Morris water maze

This test provides measures of hippocampal-dependent spatial learning and memory. A 121-cm water maze was used. Animals were given a series of three trials, ~30 min apart, per day to find a submerged platform (~1 cm below water level) in a large tank filled with water made opaque through the addition of white tempera-based nontoxic paint at 23.0° ± 1.0°C, in a room sperated from the operator by a curtain. The pool was surrounded by large panels with geometric black and white designs that serve as distal cues. Maximum trial time was limited to 60 s, whereupon mice were guided to the platform. Mice were allowed to remain on the platform for 5 s and then were gently towel-dried and moved to their home cage under a heating lamp until dry. At the end of training, a probe trial was conducted where the platform was unavailable to measure the retention of the former platform location. The time each animal spent in the quadrant formerly containing the platform and the number of passes over that location provided a measure of memory.

Closed arm Y maze

Spatial memory is assessed by placing animals in a Y-shaped maze with one arm seal off. The maze is made of white Plexiglas with three arms, with equal angles between all arms distinct shapes were placed at

the end arm of the maze. Each animal is placed in an arm of the maze and allowed to acclimate for a 5 minutes period. The mouse was then returned to its cage. After 4hr the mouse was returned to the maze with all 4 arms open. Latency to the previously sealed arm was recorded.

Rotarod

Animals were placed and allowed to acclimate on the rotarod under gentle movement at 5 rpm. Once acclimated a timer was started and rotarod speed increased to a maximal speed of 40 rpm. The latency to fall was recorded. A training session was performed on the first day with a trial session on the following day.

Grip strength assay

Mice were gently grasped by the base of their tail and allowed it to grip the bar of the grip strength meter with its forepaws. The mouse was then slowly and steadily pulled backwards until it released the bar.

Treadmill assay

Mice per placed on the treadmill and allowing them to acclimate for 1 minute. Then, shock was turned on at 1 Hz with 0.5 intensity. The treadmill was started at a speed of 5 m/min and set it to accelerate at 1 m/min. Animals were monitored. If a mouse reached the shock zone it was nudged back onto the treadmill. If it remained at the end of the treadmill for more than 5 seconds it was removed and the time and speed recorded.
